# Blood Vessel, VEGF, and FGF Expression in Traumatic Ulcer Healing of Wister Rats after Application of Chitosan- *Aloe vera* Gel

**DOI:** 10.1055/s-0045-1810118

**Published:** 2025-08-01

**Authors:** Sularsih Sularsih, Yasmin Nabila Athaya, Fitria Rahmitasari, Dwi Setianingtyas, Nur Tsurayya Priambodo, Wibi Riawan

**Affiliations:** 1 Department of Dental Materials, Faculty of Dentistry, University of Hang Tuah, Surabaya, Indonesia; 2 Department of Oral Medicine, Faculty of Dentistry, University of Hang Tuah, Surabaya, Indonesia; 3 Department of Biochemistry and Biomolecular, Faculty of Medicine, Brawijaya University, Jawa Timur, Indonesia

**Keywords:** chitosan, *Aloe vera*, traumatic ulcer healing, blood vessel, VEGF, FGF

## Abstract

**Objectives:**

The purpose of this study was to analyze the application of chitosan (CH)- *Aloe vera* (AV) gel on blood vessels, vascular endothelial growth factor (VEGF), and fibroblast growth factor (FGF) expression in traumatic ulcer healing.

**Materials and Methods:**

Chitosan powder with deacetylation degree of 93% dissolved in 2% acetic acid, neutralized with NaOH solution, and combined with AV gel. Traumatic ulcers were made with a 4-mm diameter along the lower labial mucosa of Wistar rats using a heated burnisher. Negative control group (KA) was treated with placebo, positive control group (KB) using 0.2% hyaluronic acid, treatment group using chitosan (CH) gel (PA) and AV gel (PB), and group using combination CH-AV gel (PC). The blood vessel numbers were analyzed with hematoxylin eosin staining and the expression of VEGF and FGF were analyzed using immunohistochemistry on days 3 and 7.

**Statistical Analysis:**

One-way analysis of variance was conducted to analyze the difference between groups and the Tukey honestly significant difference multiple comparisons test was used to analyze different pairs of groups ( *p* < 0.05).

**Results:**

Blood vessels, VEGF, and FGF expression were higher in the treatment group using combination CH-AV gel on days 3 and 7 compared with the control group and treatment group using CH or AV gel ( *p* < 0.05).

**Conclusion:**

The application of CH-AV gel increased the number of blood vessels, VEGF, and FGF expression in traumatic ulcer healing of Wistar rats.

## Introductions

Mechanical trauma due to denture, orthodontic brackets, or chemical agents includes dental material used by dentist; thermal irritation and radiation can cause common lesion of traumatic ulcer. The damage of oral epithelial tissue of traumatic ulcer causes discomfort, pain, and interferes with chewing or speaking. Effective treatment of traumatic ulcers requires both pain management and accelerated wound healing. 1 2 The topical treatment of traumatic ulcer such as hyaluronic acid gel is reported to cause allergic reaction and hypersensitivity. Application of steroid gel causes resistance and unpleasant side effects. 2 3 Herbal and natural marine resources therapies were studied because of their minimal side effects. 4 5 Chitosan is a very unique marine polysaccharide considered as an antibacterial, nonallergenic, nontoxic, biocompatible, bioabsorbable, and biodegradable agent. Chitosan has angiogenic. Antioxidant, and anti-inflammatory activities. 6 7 Chitosan with degree of deacetylation greater than 70% has internal hydrogen bonding which can potentially provide better surface and film forming properties. 6 In a previous study, 1% chitosan gel accelerated traumatic ulcer healing by increasing the density of collagen fiber. 8 Chitosan stimulates macrophage and promotes transforming growth factor beta-1 (TGF-β1) and tumor necrosis factor-alpha expression, which play a role in the regulation of inflammatory responses. 8 9 *Aloe vera* is a traditional medicine plant considered as a natural healer. The bioactive components in *Aloe vera* , including polysaccharides, vitamins, amino acids, enzymes, and minerals, have antifungal, antivirus, antibacterial, antiseptic, inflammatory, antioxidant, and wound healing properties. 10 The combination of chitosan and *Aloe vera* can provide synergistic effect on the wound healing process. It promotes cell migration, proliferation, and angiogenesis. 11 12

The phases of the traumatic ulcer healing process consist of inflammation, proliferation, and remodeling. Growth factor such as vascular endothelial growth factor (VEGF) and fibroblast growth factor (FGF) are the first growth factors released by macrophages, fibroblast, and endothelial cells to initiate the wound healing process. VEGF plays a crucial role in the formation of new blood vessels, promoting revascularization, and inducing vascular permeability in injured tissues. 2 4 13 The formation of new blood vessels is essential for the healing process, as it facilitates the delivery of oxygen, nutrients, and healing mediators to support cellular proliferation. Angiogenesis plays a vital role in the healing process of traumatic ulcers, and VEGF and FGF are key regulators of this process. VEGF promotes angiogenesis, while FGF regulates cell migration, proliferation, and granulation of tissue formation. 3 14 The acceleration of traumatic healing was observed in a number of blood vessels, VEGF, and FGF expression. The study aims to investigate the application of chitosan- *Aloe vera* gel on the number of blood vessels, VEGF, and FGF expression in traumatic ulcer healing of Wistar rats.

## Materials and Methods

### Preparation of Chitosan and *Aloe vera* Gel

Chitosan powder was obtained from tiger prawn shells through a multistep homemade process involving deproteinization, depigmentation, and deacetylation resulting in chitosan powder with a 93% deacetylation degree. A chitosan gel was prepared by dissolving 1 g of chitosan powder, synthesized from tiger prawn shells, in 100 mL of 2% acetic acid (Merck). The mixture was stirred, centrifuged, and neutralized with NaOH solution (Merck) before being filtered. 15

*Aloe vera* gel was extracted from fresh *Aloe vera* leaves obtained from Batu, Malang, East Java, Indonesia. The gel was blended, freeze-dried at a temperature of 4°C and powdered. The powder was then dissolved in 70% ethanol and mixed with sodium carboxymethyl cellulose (Na-CMC) (Merck) to create a 50% *Aloe vera* gel. This gel was then mixed with chitosan gel in a 1:1 ratio and neutralized with NaOH solution (Merck) to form a chitosan- *Aloe vera* gel. 9 15

### *In Vivo* Study

The present study was an experimental research conducted on male *Rattus norvegicus* aged 2 to 3 months, with body weights ranging from 150 to 200 g. The rats were sourced from the experimental animal laboratory at Faculty of Dentistry, University of Hang Tuah, Surabaya, Indonesia. The study protocol ethics approval was obtained from the Faculty of Dentistry, Ethical Committee with registration number 012/KEPK-FKGUHT/X/2023. Experimental animals were conducted in the Animal Testing Laboratory, Faculty of Dentistry. The rats were kept in collective cages with room temperature (27°C) with free access to water and standard diet. The rats were anesthetized using ketamine 10% injection (Kepro Pharmacy, Holland) and xylazine 2% injection (Xyla, Interchemie, Netherland). Ulcers with diameter of 4 mm were formed 24 hours after a burnisher was heated on the lower labial mucosa of rats for 3 seconds. Rats were randomly assigned to five groups with five samples in each group, and observed for 3 and 7 days. 4 5 The negative control group was treated with placebo (KA), the positive control group with 0.2% hyaluronic acid (KB), the treatment group using chitosan (CH) gel (PA), using *Aloe vera* (AV) gel (PB), and the final group using combination of chitosan- *Aloe vera* (CH-AV) gel (PC).

At 3 and 7 days posttreatment, rats were sacrificed, and the lower labial mucosal tissues, including the ulcerated areas, were harvested and fixed in 10% formalin buffer. The tissues were then embedded in paraffin blocks, cut into 5-μm thick sections. The specimens were made and stained with hematoxylin eosin (HE; Sigma Aldrich) to observe the number of blood vessel formation. The immunohistochemical examination of VEGF expression was performed using VEGF mouse antibody monoclonal (Santa Cruz Biotechnology Inc, United States) and FGF expression using FGF mouse antibody monoclonal (Santa Cruz Biotechnology Inc, United States). The number of blood vessels, VEGF, and FGF expression was observed on days 3 and 7. Histological preparations were examined using a light microscope (Nikon H600L) at magnifications of 40 × , 400 × , and 1000 × .

### Statistical Analysis

The obtained data of the number of blood vessels, VEGF, and FGF expression were analyzed for normality using the Shapiro–Wilk test, revealing a normal distribution ( *p* > 0.05). Homogeneity of variance was confirmed using the Levene test ( *p* > 0.05). A one-way analysis of variance was performed to compare differences between groups, followed by the least significant difference test to determine significant differences ( *p* < 0.05).

## Results

### The Number of Blood Vessels in Traumatic Ulcer Healing

**Fig. 1:**
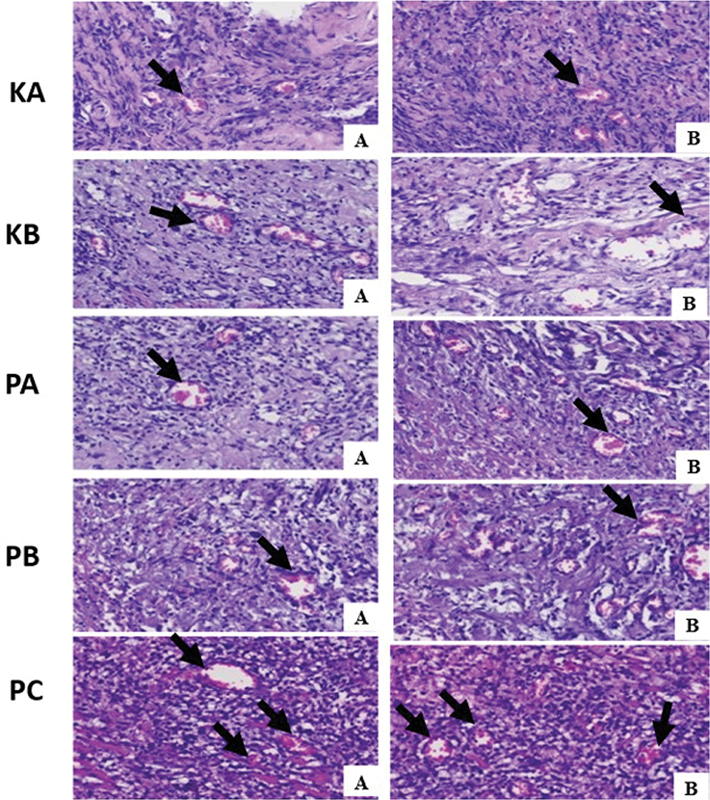
Blood vessels in traumatic ulcer tissue in each group were analyzed with hematoxylin eosin staining (black arrow). Magnification at 400 × . ( **A** ) On day 3. ( **B** ) On day 7.

Histopathological examination with HE staining at 400× magnification revealed the number of blood vessels on days 3 and 7. As shown in Fig. 1 , the highest number of blood vessels was observed in the PC group treated with combination of chitosan- *Aloe vera* (CH-AV) gel, compared to the control group and other treatment groups (PA and PB). In contrast, the lowest number of blood vessels was found in the KA control group ( Table 1 ). In the PA and PB groups, there were more blood vessels than the KA control group (placebo) and KB control group using hyaluronic acid gel.

**Table 1:** The average number of blood vessel, VEGF, and FGF expression on days 3 and 7 in oral traumatic ulcer

Groups	Blood vessel			VEGF			FGF		
	3 d	7 d	*p* -Value	3 d	7 d	*p* -Value	3 d	7 d	*p* -Value
KA	0.60 ± 0.55 a	1.00 ± 0.00 a	0.009 e	3.00 ± 1.59 a	3.60 ± 1.14 a	1.000	2.40 ± 1.67 a	4.40 ± 1.14 a	0.362
KB	1.60 ± 0.54 b	2.00 ± 0.00 b	0.009 e	6.60 ± 1.52 b	6.90 ± 1.50 b	1.000	4.00 ± 1.58 b	5.60 ± 1.10 b	1.000
PA	1.80 ± 0.45 b	2.60 ± 0.55 c	0.002 e	6.40 ± 2.07 b	7.60 ± 1.14 c	0.975	6.20 ± 1.30 c	7.60 ± 1.67 c	0.887
PB	2.20 ± 0.45 c	2.24 ± 0.47 bc	0.000 e	7.00 ± 1.58 c	8.00 ± 1.59 c	0.993	7.40 ± 1.52 c	8.60 ± 1.14 c	0.948
PC	3.60 ± 0.54 d	5.00 ± 0.02 d	0.007 e	9.20 ± 2.39 d	11.40 ± 1.52 d	0.047 e	8.60 ± 1.14 d	10.85 ± 2.00 d	0.043 e

Abbreviations: FGF, fibroblast growth factor; VEGF, vascular endothelial growth factor.

Note: Negative control group (KA) was treated with placebo, positive control group (KB) using 0.2% hyaluronic acid, treatment group using chitosan (CH) gel (PA) and AV gel (PB), and group using combination CH-AV gel (PC).

a,b,c,d Significant differences between groups are indicated by different superscripts as determined by the Tukey honestly significant difference (HSD) multiple comparison test.

e *p* < 0.05.

The Tukey honestly significant difference (HSD) multiple comparison test result is shown in Table 1 . Significant differences of blood vessels in the PA, PB, and PC groups compared to KA on days 3 and 7 are shown in Table 1 . There were significant differences between the PC group and the KA, KB, PA, and PB groups in the number of blood vessels for days 3 and 7 with Tukey HSD multiple comparisons test showing a value of *p* = 0.000 ( *p* < 0.05).

### The VEGF Expression in Traumatic Ulcer Healing

**Fig. 2:**
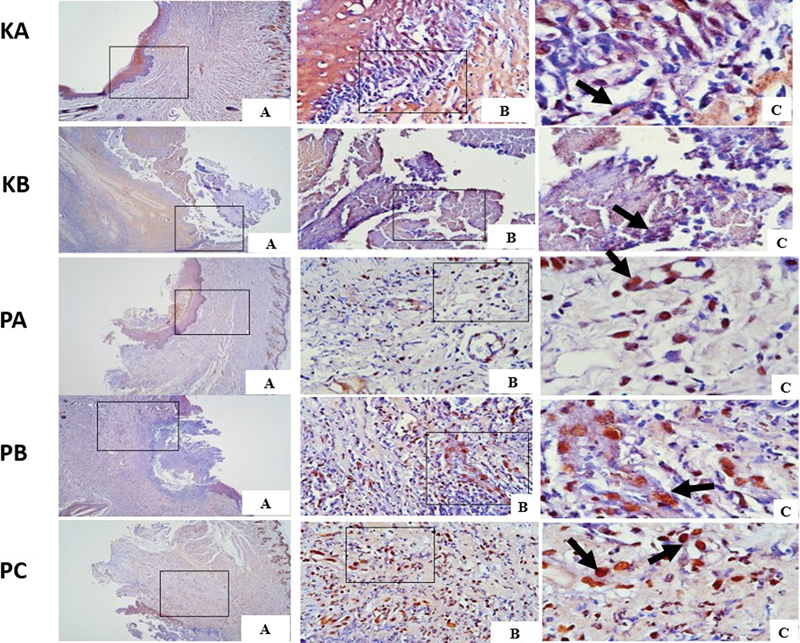
Vascular endothelial growth factor (VEGF) expression in traumatic ulcer tissue on day 3 in each group with immunohistochemistry (black arrow). ( **A** ) Magnification 40 × . ( **B** ) Magnification 400 × . ( **C** ) Magnification 1000 × .

**Fig. 3:**
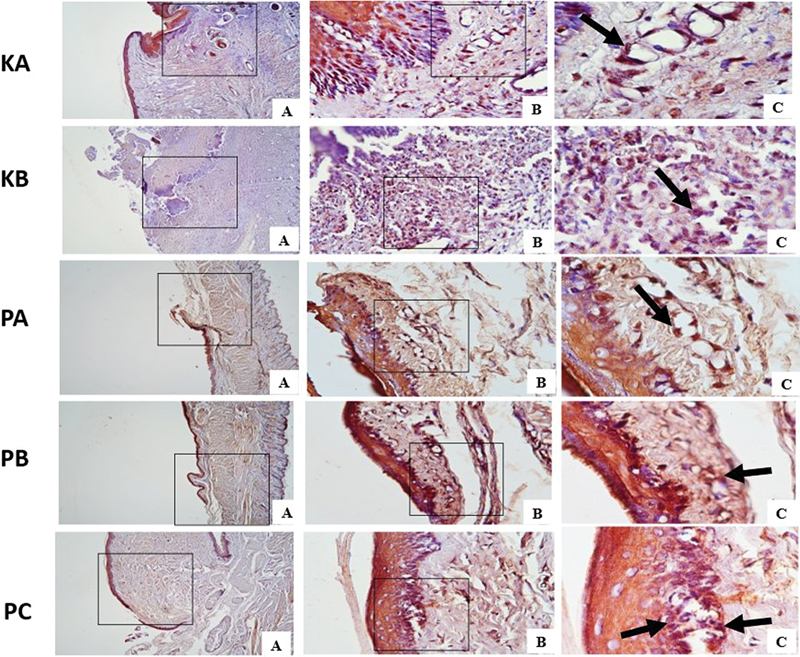
Vascular endothelial growth factor (VEGF) expression in traumatic ulcer tissue on day 7 in each group with immunohistochemistry (black arrow). ( **A** ) Magnification 40 × . ( **B** ) Magnification 400 × . ( **C** ) Magnification 1000 × .

VEGF expression on days 3 and 7 was observed on immunohistochemistry examination at 40 × , 100 × , and 400× magnification of light microscope as shown in Figs. 2 and 3 . VEGF expression was evaluated by counting the brown immunoreactive cells, which represented cytoplasmic staining of epithelial cells, indicating a positive reaction between VEGF antigen and monoclonal VEGF antibody. The highest expression of VEGF on days 3 and 7 of traumatic ulcer healing was observed in the PC group compared to the control group and other treatment groups (PA and PB group). The lowest expression of VEGF was observed in KA control group ( Table 1 ). The PC group treated with CH-AV gel showed the highest VEGF expression during traumatic ulcer healing followed by the PB, PA, KB, and KA groups, respectively.

The results of the multiple comparison HSD Tukey test presented in Table 1 revealed significant differences in VEGF expression between the PA, PB, and PC groups compared to the KA group on days 3 and 7. Furthermore, the results showed that, while there were no significant differences in VEGF expression between the KA, KB, PA, and PB groups at days 3 and 7, the PC group exhibited a significant difference. VEGF expression continued to increase on day 7 in all groups.

### The Expression of FGF in Traumatic Ulcer Healing

**Fig. 4:**
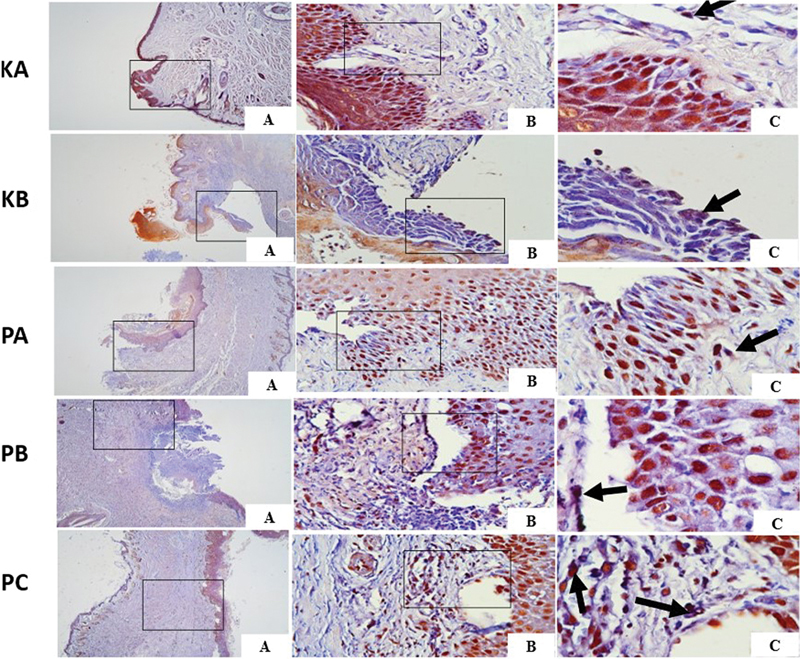
Fibroblast growth factor (FGF) expression in traumatic ulcer tissue on day 3 in each group with immunohistochemistry (black arrow). ( **A** ) Magnification 40 × . ( **B** ) Magnification 100 × . ( **C** ) Magnification 1000 × .

**Fig. 5:**
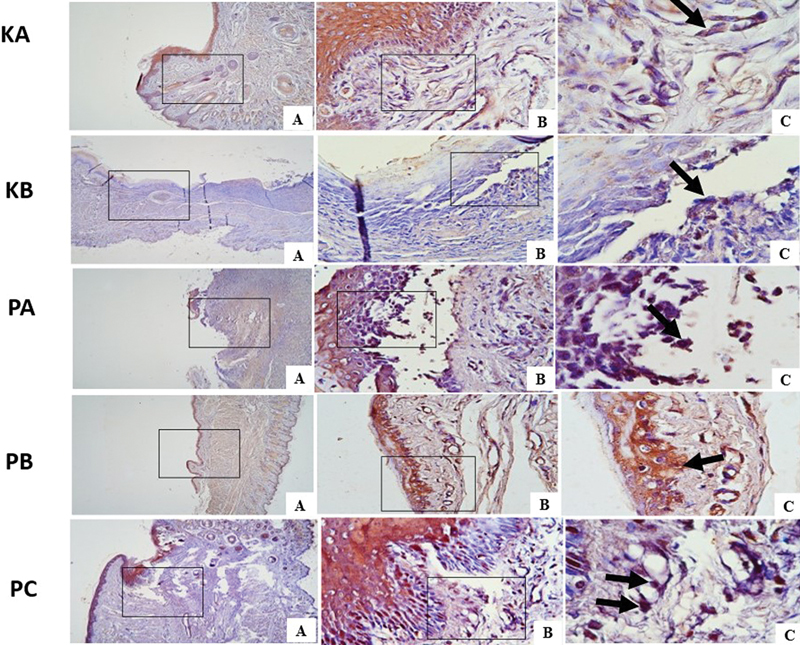
Fibroblast growth factor (FGF) expression in traumatic ulcer tissue on day 7 in each group with immunohistochemistry (black arrow). ( **A** ) Magnification 40 × . ( **B** ) Magnification 100 × . ( **C** ) Magnification 1000 × .

The expression of FGF on days 3 and 7 was observed on immunohistochemistry examination at 40 × , 100 × , and 400× magnification under a light microscope as shown in Figs. 4 and 5 . Data on positive expression of FGF were evaluated by counting the brown immunoreactive in fibroblast cells, which indicates a positive reaction between FGF antigen and monoclonal FGF antibody. The highest expression of FGF on days 3 and 7 of traumatic ulcer healing was observed in the PC group compared to the control group and other treatment groups (PA and PB groups). The lowest expression of FGF was observed in the KA control group ( Table 1 ). FGF expression continued to increase on day 7 in all groups.

As shown in Table 1 , the Tukey HSD test results indicated significant differences in VEGF expression between the PA, PB, and PC groups and the KA and KB groups on days 3 and 7. In contrast, no significant differences in the number of blood vessels were found between the PA and PB groups on days 3 and 7. Furthermore, the results revealed that the treatment duration at 3 and 7 days had no significant impact on VEGF expression in the KA, KB, PA, and PB groups, whereas the PC group exhibited a significant difference.

## Discussion

Traumatic ulcer healing process involves hemostasis, inflammation, proliferation, and remodeling phase. Under normal conditions, the traumatic ulcer healing process needs approximately 48 hours. However, today, herbal and natural agents can be used to accelerate ulcer healing. The molecular activation process requires involving activation of platelets, keratinocytes, fibroblast, endothelial cells, and macrophage. These cells have an important role in migration and proliferation. Some cellular responses in ulcer healing are regulated by these cells. 13 14 16

In the proliferation phase, keratinocytes migrate to repair the injured areas during the epithelialization process. Blood vessels play a vital role in the repair of injured areas by supplying collagen to the new extracellular matrix and providing oxygen, various nutrients, cytokines, and immunocompetent cells supporting the proliferation phase. 3 13 17 Growth factors such as TGFβ, platelet-derived growth factor (PDGF), VEGF, and FGF play a role in the migration, proliferation, and formation of granulated tissue of ulcer healing. 4 13 18 As an angiogenic growth factor, VEGF plays a vital role in inducing the formation of permeable blood vessels, enhancing vascular permeability, and stimulating endothelial cell proliferation and differentiation, thereby promoting angiogenesis. FGF triggers angiogenesis, anti-inflammatory process, and fibroblast proliferation. 4

In our study, gel formulation was used for application on ulcer healing. Gel is necessary to consider as an ideal formulation form for ulcer healing. Gel has a strong tissue-adhesive property and is stable so as to provide a longer working time and has the ability for absorption. 19 In this study, blood vessels and the expression of VEGF and FGF were significantly upregulated in the treatment groups treated with CH, AV, and CH-AV gel, compared to the control group. Chitosan (CH) is a promising marine polysaccharide for medical research. It has a variety of biological properties with good biocompatibility, biodegradability of biopolymer agent, anti-inflammation, and antibacterial. CH is derived from N-deacetylation from chitin. It has strong positive electrical charge which strongly bonds to negative charge. CH gel adheres to injury site or wound via electrostatic interaction due to its cationic characteristic. 19 20 Active N-acetyl-D-glucosamine of CH cross-link with glycosaminoglycan and glycoproteins and activates macrophages releasing important growth factors such as TGF-β1, PDGF, VEGF, and FGF. 20 Previous studies have reported anti-inflammation activity of CH. It modulates macrophage switch from M1 to M2 polarization. M2 secretes growth factors including TGF-β1, FGF, VEGF, PDGF, and collagen type 1, which contribute to fibroblast proliferation and collagen production. The increasing of VEGF and FGF stimulates angiogenesis, including new blood vessels, thus accelerating the ulcer healing process. 6 21

Blood vessels, VEGF, and FGF expression in the treatment group using AV gel in this study were higher than the control group using hyaluronic acid gel. Naturally and biologically active compounds of *Aloe vera* such as amino acids, polysaccharides mannan, vitamin, alkaline phosphatase and bradikinase enzyme, anthraquinones, auxin, saponins, flavonoids, and minerals have been reported to have wound healing properties. *Aloe vera* has anti-inflammatory, antibacterial, and antivirus activity. 10 11 Anthraquinones components, namely, barbaloin, emodin, and alaoin, have proven to have strong anti-inflammatory effects. The bioactive compounds of *Aloe vera* can reduce inflammation so it can increase the number of blood vessels and VEGF and FGF expression. Glucomannan, a mannose-rich polysaccharide component, interacts with the growth factor receptor of fibroblast and stimulates its activity and proliferation. 10 Thus, in this study, the treatment group using *Aloe vera* was found to stimulate proliferation and enhance fibroblast activation with increasing FGF expression.

The amino groups present in chitosan, a cationic polysaccharide obtained from the deacetylation of chitin in crustaceans, are responsible for its key properties including its ability to adhere to mucous membranes. Some previous studies suggested the combination of CH-AV gel significantly increases physicochemical, mechanical, mucoadesive, roughness, wettability, and color properties. 10 Its combination was found to promote high cell viability in *in vitro* cell culture studies, as confirmed by the results of MTT assay testing. 22 In our study, the number of blood vessels and VEGF and FGF expression in the treatment group with CH-AV gel was higher compared to the control and treatment groups with CH gel or AV gel. The combination of CH-AV has a synergistic anti-inflammatory effect and ability to inhibit nuclear factor-kappa B (NF-kB) activation in macrophage. 9 12 The inhibiting of NF-kB activation resulted in more dominant M2 polarization. The role of M2 macrophages in the formation of new blood vessels and VEGF expression on days 3 and 7 after CH-AV gel treatment is critical. VEGF promotes the formation of permeable blood vessels and enhances vascular permeability during the initial stages of traumatic ulcer healing, stimulating cell proliferation and differentiation during angiogenesis. The resulting blood vessels provide oxygenated blood and nutrients, facilitating cell activity during the proliferation phase and enhancing traumatic ulcer healing. 13 In our study, FGF expression increased after CH-AV gel treatment. FGF plays a role in angiogenesis and anti-inflammatory process on days 3 and 7. The proliferation of fibroblast in traumatic ulcer healing occurs on day 3 and reaches a peak on days 7 until 14. The increase in FGF increases the proliferation of fibroblast cells. Fibroblast cells are most commonly cells in connective tissue and a source of extracellular matrix. FGF also induced collagen synthesis, which plays a key role in ulcer healing. 4 13 14 Therefore, the application of CH-AV gel can improve the traumatic ulcer healing process, increasing the number of blood vessels and VEGF and FGF expression. Further studies are needed on the molecular level or proangiogenic genes or other proteins needed to accelerate the traumatic ulcer healing process.

## Conclusion

It could be concluded that the application of chitosan- *Aloe vera* gel accelerates traumatic ulcer healing by increasing the number of blood vessels and VEGF and FGF expression. The combination of chitosan- *Aloe vera* gel has potential as herbal and natural therapies in the treatment of oral ulcer.

## References

[1] Gilvetti C Porter S R Fedele S Traumatic chemical oral ulceration: a case report and review of the literature Br Dent J 2010 208 07 297 300 20379246 10.1038/sj.bdj.2010.295

[2] Arundina I Diyatri I Kusumaningsih T Surboyo M DC Monica E Afanda N M The role of rice liquid smoke in traumatic ulcer healing Eur J Dent 2021 15 01 33 38 32777835 10.1055/s-0040-1714445 PMC7902118

[3] Kusumaningsih T Irmawati A Ernawati D S Prahasanti C Aljunaid M Amelia S The differences in the number of fibroblasts and blood vessels after the topical and systemic administration of *Lactobacillus casei* Shirota probiotics for the treatment of traumatic ulcers in Wistar rats ( *Rattus norvegicus* ) Vet World 2021 14 05 1279 1283 34220131 10.14202/vetworld.2021.1279-1283 PMC8243686

[4] Puspasari A Harijanti K Soebadi B Hendarti H T Radithia D Ernawati D S Effects of topical application of propolis extract on fibroblast growth factor-2 and fibroblast expression in the traumatic ulcers of diabetic *Rattus norvegicus* J Oral Maxillofac Pathol 2018 22 01 54 58 29731557 10.4103/jomfp.JOMFP_82_17 PMC5917542

[5] Sari R P Larashati D ID Aldiana C Nafi'ah N Damaiyanti D W Kurniawati A Aplication of stichopus hermanni nanoparticle gel in the healing of traumatic ulsers Eur J Dent 2023 17 02 330 336 36690026 10.1055/s-0042-1759884 PMC10329517

[6] Periayah M H Halim A S Saad A Z Chitosan: a promising marine polysaccharide for biomedical research Pharmacogn Rev 2016 10 19 39 42 27041872 10.4103/0973-7847.176545 PMC4791986

[7] Vagropoulou P Trentsiou M Georgopoulou A Hybrid chitosan/gelatin/ nanohydroxyapatite scaffolds proote odontogenic differentiation of dental pulp stem cells and in vitro biomineralization Dent Mater J 2021 37 01 23 36 10.1016/j.dental.2020.09.021 33208264

[8] Sularsih S Suhartono M Nafi'ah The differences influnces application combination of chitosan with high and low molecular weight - Aloe vera gel to collagen density in traumatic ulcer healing Dent J 2018 12 01 60 71

[9] Sularsih S The application of chitosan with different molecular weight to expression of tumor necrosis factor alpha in dental extraction healing of Rattus norvegicus JMKG 2016 15 01 15 22

[10] Rahman S Carter P Bhattarai N *Aloe Vera* for tissue engineering applications J Funct Biomater 2017 8 01 1 17 28216559 10.3390/jfb8010006 PMC5371879

[11] Kresnoadi U Rahayu R P Rubianto M Sudarmo S M Budi H S TLR2 signaling pathway in alveolar bone osteogenesis induced by *Aloe vera* and xenograft (XCB) Braz Dent J 2017 28 03 281 286 29297547 10.1590/0103-6440201600834

[12] Sularsih S Mulawarmanti D Rahmitasari F Siswodihardjo S In silico analysis of glycosaminoglycan-acemannan as a scaffold material on alveolar bone healing Eur J Dent 2022 16 03 643 647 35453170 10.1055/s-0041-1736592 PMC9507609

[13] Arundina I Diyatri I Surboyo M DC Monica E Afanda N M Growth factor stimulation for the healing of traumatic ulcers with liquid rice hull smoke J Taibah Univ Med Sci 2021 16 03 431 439 34140871 10.1016/j.jtumed.2021.01.003 PMC8178683

[14] Ayuningtyas N F Hendarti H T Soebadi B Expression of VEGF and CD-31 in traumatic ulcer of diabetic Wistar rats after application of *Citrus limon* peel essential oil J Oral Biol Craniofac Res 2023 13 03 380 385 37025967 10.1016/j.jobcr.2023.03.009 PMC10070900

[15] Sularsih The pore size of chitosan-aloe vera scaffold and its effect on VEGF expressions and woven bone alveolar healing of tooth extraction of cavia cobaya Dent J 2020 53 03 115 121

[16] Surboyo M DC Mahdani F Y Ernawati D S Sarasati A Rezkita F The macrophage responses during diabetic oral ulser healing by liquid coconut shell smoke: an immunohistochemical analysis Eur J Dent 2020 14 03 410 414 32447753 10.1055/s-0040-1712776 PMC7440958

[17] Surboyo M DC Arundina I Rahayu R P Mansur D Bramantoro T Potential of distilled liquid smoke derived from coconut (Cocos nutrifera L) shell for traumatic ulcer healing in diabetic Rats Eur J Dent 2019 13 02 271 279 31487751 10.1055/s-0039-1693527 PMC6777171

[18] Sularsih S Fransiska W Salsabila S Rahmitasari F Soesilo D Prananingrum W Potency of the combination of chitosan and hydroxyapatite on angiogenesis and fibroblast cell proliferation in direct pulp capping of Rattus norvegicus Eur J Dent 2024 18 04 1135 1141 38698616 10.1055/s-0044-1782212 PMC11479727

[19] Rahmani F Larbi Bouamrane O Ben Bouabdallah A Atanase L I Hellal A Apintiliesei A N Biomimetic hydroxyapatite crystal growth on phosphorylated chitosan films by in vitro mineralization used dental substitute material Polymers (Basel) 2023 15 11 2470 2492 37299269 10.3390/polym15112470 PMC10255572

[20] Putranto A W Suprastiwi E Meidyawati R Agusnar H Characterization of novel cement-based carboxymethyl chitosan/amorphous calcium phosphate Eur J Dent 2022 16 04 809 814 35016237 10.1055/s-0041-1739449 PMC9683891

[21] Szulc M Lewandowska K Biomaterials based on chitosan and its derivatives and their potential in tissue engineering and other biomedical application-a review Molecules 2022 28 01 247 264 36615441 10.3390/molecules28010247 PMC9821994

[22] Sularsih S Rahayu R P Cytotoxicity of combination chitosan with different molecular weight and ethanol extracted aloe vera using MTT assay IOP Conf Ser Earth Environ Sci 2018 217 12030

